# Haplotype-specific chromatin looping reveals genetic interactions of regulatory regions modulating gene expression in 8p23.1

**DOI:** 10.3389/fgene.2022.1008582

**Published:** 2022-09-07

**Authors:** Mariana Saint Just Ribeiro, Pulak Tripathi, Bahram Namjou, John B. Harley, Iouri Chepelev

**Affiliations:** ^1^ Center for Autoimmune Genomics and Etiology, Cincinnati Children’s Hospital Medical Center, Cincinnati, OH, United States; ^2^ Research Service, US Department of Veterans Affairs Medical Center, Cincinnati, OH, United States; ^3^ Cincinnati Education and Research for Veterans Foundation, Cincinnati, OH, United States

**Keywords:** gene regulation, enhancer, enhancer-like promoter, genetic interaction, missing heritability, *BLK*, systemic lupus erythematosus (SLE), *FAM167A*

## Abstract

A major goal of genetics research is to elucidate mechanisms explaining how genetic variation contributes to phenotypic variation. The genetic variants identified in genome-wide association studies (GWASs) generally explain only a small proportion of heritability of phenotypic traits, the so-called missing heritability problem. Recent evidence suggests that additional common variants beyond lead GWAS variants contribute to phenotypic variation; however, their mechanistic underpinnings generally remain unexplored. Herein, we undertake a study of haplotype-specific mechanisms of gene regulation at 8p23.1 in the human genome, a region associated with a number of complex diseases. The *FAM167A-BLK* locus in this region has been consistently found in the genome-wide association studies (GWASs) of systemic lupus erythematosus (SLE) in all major ancestries. Our haplotype-specific chromatin interaction (Hi-C) experiments, allele-specific enhancer activity measurements, genetic analyses, and epigenome editing experiments revealed that: 1) haplotype-specific long-range chromatin interactions are prevalent in 8p23.1; 2) *BLK* promoter and *cis*-regulatory elements cooperatively interact with haplotype-specificity; 3) genetic variants at distal regulatory elements are allele-specific modifiers of the promoter variants at *FAM167A-BLK*; 4) the *BLK* promoter interacts with and, as an enhancer-like promoter, regulates *FAM167A* expression and 5) local allele-specific enhancer activities are influenced by global haplotype structure due to chromatin looping. Although systemic lupus erythematosus causal variants at the *FAM167A-BLK* locus are thought to reside in the *BLK* promoter region, our results reveal that genetic variants at distal regulatory elements modulate promoter activity, changing *BLK* and *FAM167A* gene expression and disease risk. Our results suggest that global haplotype-specific 3-dimensional chromatin looping architecture has a strong influence on local allelic *BLK* and *FAM167A* gene expression, providing mechanistic details for how regional variants controlling the *BLK* promoter may influence disease risk.

## Introduction

Contemporary genetics research aims to understand mechanisms of how variation in DNA sequence underlies phenotypic variation in normal and disease states. Genome-wide association studies (GWASs) have identified thousands of genetic loci associated with over 4,000 phenotypes (www.ebi.ac.uk/gwas/). However, genetic variants identified in GWAS studies explain only a modest fraction of total genetic risk in complex diseases, leading to the so-called missing heritability problem (Manolio et al., 2009). Recent evidence suggests that additional common variants beyond the lead GWAS single nucleotide polymorphisms (SNPs) contribute to disease risk (Gusev et al., 2013; Corradin et al., 2014; Gusev et al., 2014; Corradin et al., 2016; Boyle et al., 2017). However, mechanistic underpinnings of how additional genetic variants contribute to disease risk have not been thoroughly investigated for the vast majority of disease risk loci. Some suggest that a proper account of genetic interactions may help solve the missing heritability problem (Zuk et al., 2012). GWAS associations most often occur in non-coding regions of the genome, presumably at gene regulatory elements (GREs) (Hindorff et al., 2009), suggesting that additional risk variants will be found in GREs as well.

Transcriptional regulation is a complex process involving 3-dimensional (3D) chromatin interactions of GREs (Li et al., 2018). Combinations of genetic polymorphisms affecting various components in this process may thus alter gene expression and contribute to disease risk. The importance of 3D genome architecture for gene regulation in normal and disease states has been increasingly appreciated and is clearly a tremendous source for important new knowledge (Babu and Fullwood, 2015; Krijger and de Laat, 2016). The techniques based on chromosome conformation capture have emerged as methods of choice for mapping the 3D structure of the human genome (de Wit and de Laat, 2012). In particular, capture Hi-C, a high-throughput method to identify chromatin interactions in large genomic regions, has been used to determine high-resolution 3D genome structures at some disease-associated loci (Dryden et al., 2014; Jäger et al., 2015).

In order to investigate the role of haplotype-specific 3D chromatin structure in allelic gene expression, we have chosen the 8p23.1 region on human chromosome 8.8p23.1 contains associations with a number of complex diseases and the largest (4.5 Mb) known common DNA inversion region in humans (Salm et al., 2012; Namjou et al., 2014). The *FAM167A-BLK* locus in this region, for example, has been consistently found to be associated with systemic lupus erythematosus (SLE) GWASs in all major ancestries (International Consortium for Systemic Lupus Erythematosus Genetics (SLEGEN) et al., 2008; Hom et al., 2008; Ito et al., 2009; Suarez-Gestal et al., 2009; Sánchez et al., 2011; Castillejo-López et al., 2012; Delgado-Vega et al., 2012; Guthridge et al., 2014).

Associations with the *BLK* locus have also been identified in rheumatoid arthritis (RA), systemic sclerosis (SSc), Sjӧgren’s syndrome (SjS), Kawasaki’s disease (KD), antiphospholipid syndrome (APS) and maturity-onset diabetes of the young (MODY), thus justifying a focused mechanistic study of this locus (Hom et al., 2008; Borowiec et al., 2009; Yin et al., 2009; Gourh et al., 2010; Tsuchiya et al., 2010; Lessard et al., 2013). BLK is a signal transduction molecule that is important for sustaining the inflammatory response, including autoimmune responses. BLK probably plays an important role in early B cell development; thus, its dysregulation may result in a breakdown of peripheral self-tolerance during B cell development (Nashi et al., 2010; Simpfendorfer et al., 2012).

SLE patients have lower levels of the *BLK* gene product, a finding that is correlated with the risk alleles at the *FAM167A-BLK* locus (Hom et al., 2008). The strongest SLE association signal at the *BLK* locus is from the promoter SNP rs13277113 (Hom et al., 2008). The trans-population mapping and sequencing strategy was used in (Guthridge et al., 2014) to identify two putative SLE causal variants, rs922483 and rs1382568, at the *BLK* promoter region. The data available have led to a general consensus in the SLE genetics research community that causal variants at *FAM167A-BLK* are very likely to be in the *BLK* promoter region.

Our haplotype-specific chromatin interaction high-resolution Hi-C experiments, allele-specific enhancer activity H3K27ac measurements, genetic analyses, and epigenome editing experiments revealed that 1) haplotype-specific long-range chromatin interactions in 8p23.1 are prevalent, 2) *BLK* promoter and *cis*-regulatory elements cooperatively and haplotype-specifically interact, 3) *BLK* promoter interacts with and, as an enhancer-like promoter, regulates *FAM167A* expression, and 4) genetic variants at distal regulatory elements are allele-specific genetic modifiers of the promoter variants at *FAM167A-BLK.*

Our findings suggest a ‘risk dosage’ model whereby disease risk alleles at multiple regulatory elements at *BLK* locus synergistically decrease gene expression, thereby increasing SLE disease risk. Although SLE causal genetic variants are thought to reside in the *BLK* promoter region, we show that haplotype-specific distal genetic variants at regulatory elements modulate the effects of *BLK* promoter variants on gene expression and disease risk. More generally, our results suggest that global haplotype-specific 3-dimensional chromatin looping architecture may have a strong influence on local allelic gene expression and disease risk in SLE, as well as in other complex diseases, and provide a model for a risk dosage approach to regulatory susceptibility loci, in general.

## Results

### Haplotype-specific chromatin looping

Long-range chromatin interactions are important for transcriptional regulation of genes (Chepelev et al., 2012; Krijger and de Laat, 2016). We suspected that causal genetic variants at *FAM167A-BLK* dysregulate normal gene expression by altering chromatin looping interactions. We thus hypothesized that in B cell lines heterozygous for SLE-associated SNPs in the 8p23.1 region, 3D chromatin structures of risk and non-risk haplotypes would differ. To test this hypothesis, we prepared capture Hi-C libraries from two 1,000 Genomes EBV-infected lymphoblastoid cell lines (LCLs), NA07000 and NA07056, both heterozygous for the SLE-associated SNP rs922483 (Guthridge et al., 2014), located in the promoter of *BLK*.

**FIGURE 1:**
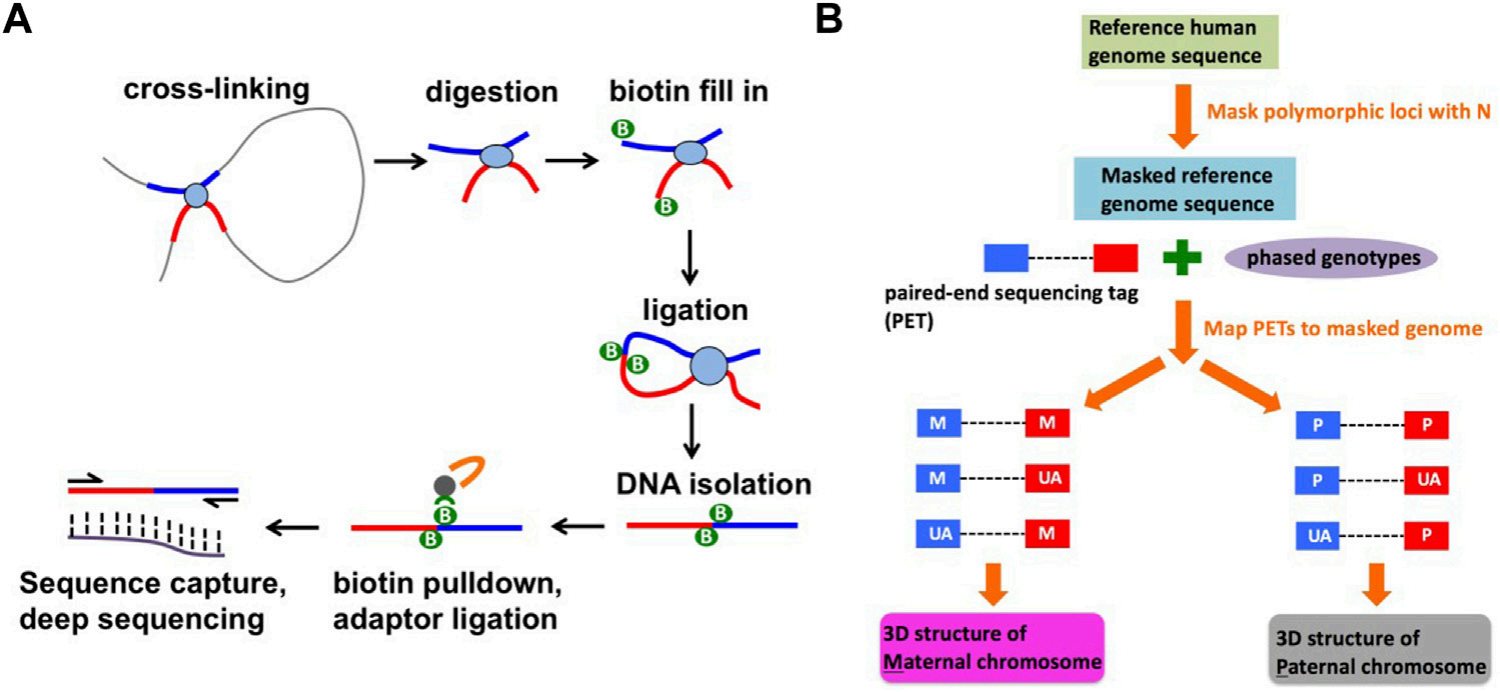
Identification of haplotype-specific chromatin interactions. **(A)** A schematic diagram of capture Hi-C method. **(B)** A computational strategy to identify haplotype-specific chromatin interactions.

**FIGURE 2:**
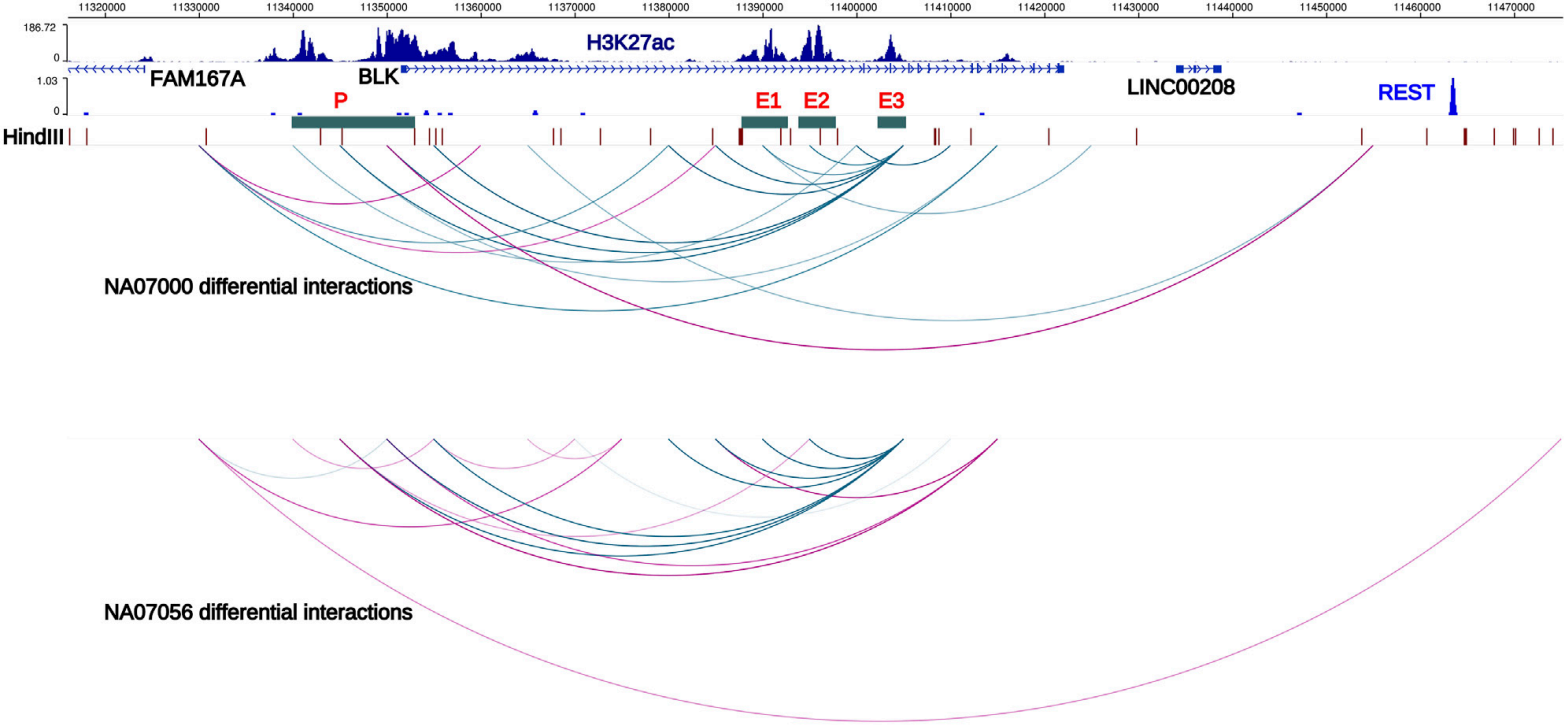
Haplotype-specific chromatin interactions at *FAM167A-BLK* locus. Arcs represent haplotype-specific chromatin interactions from our Hi-C data in NA07000 and NA07056 LCLs. Blue arcs depict long-range chromatin interactions which are weaker on risk haplotype. Purple arcs depict interactions that are stronger on the risk haplotype than on the non-risk haplotype. Interaction of *BLK* promoter with the enhancer E3 is weaker and with the REST repressor binding region is stronger on SLE risk haplotype, consistent with reduced expression of *BLK* on the risk haplotype. Interaction of enhancer E3 with enhancers E1 and E2 is weaker on the risk haplotype. Also displayed are the H3K27ac signals in NA12878 LCLs (ENCODE data) and HindIII restriction sites. E1, E2, E3; putative enhancers 1, 2, and 3. P; *BLK* promoter, locus of SLE credible set BLK-CS-11 (Supplementary Table S5).

Capture Hi-C is a high-throughput, cost-effective method to identify long-range 3D chromatin interactions in a subset of the genome (Dryden et al., 2014) (see Materials and Methods, and Figure 1). We identified the haplotype-resolved 3D structure of a 3 Mb region in 8p23.1 at 5 kb resolution. At the false discovery rate (FDR) of 5%, we have identified 780 and 791 differential haplotype-specific chromatin interactions in NA07000 and NA07056 cell lines, respectively (Supplementary Tables S1, S2). Approximately half of these interactions are stronger and half of the interactions are weaker on SLE risk haplotype than on non-risk haplotype. Among the differential haplotype-specific interactions in 8p23.1, some are in the vicinity of the *BLK* locus (Figure 2).

In the ENCODE Project (encodeproject.org) data, several extended regions at the *BLK* locus show enrichment for the H3K27ac histone modification mark in LCLs and primary B cells. (We designate these regions E1, E2, E3, and P in Figure 2). The H3K27ac mark is thought to be associated with active enhancers in the genome (Creyghton et al., 2010). Our haplotype-specific Hi-C data described below strongly suggest that these regions positively regulate *BLK* expression. Our capture Hi-C data show that enhancer E3, located more than 50 kb away from the *BLK* transcription start site, interacts with the promoter in a haplotype-specific manner. Consistent with lower expression of *BLK* on the SLE risk haplotype, P-E3 interaction frequency on the SLE risk haplotype is lower than on the non-risk haplotype (Figure 2). It has been known for some time that enhancers can interact with other enhancers in the 3D genome (Chepelev et al., 2012). We found that enhancers also interact with each other in a haplotype-specific way (Figure 2), again with weaker interactions on the risk haplotype. These results are consistent with long-range chromatin interactions between enhancers playing a role in co-operatively enhancing *BLK* expression.

Intriguingly, we found that the interaction of the *BLK* promoter with a distal REST binding site located 115 kb away (Figure 2) is stronger on SLE risk haplotype, consistent with the repressive role of REST (repressor element 1-silencing transcription factor) (Ooi and Wood, 2007) and lower expression level of *BLK* gene on SLE risk haplotype.

**FIGURE 3:**
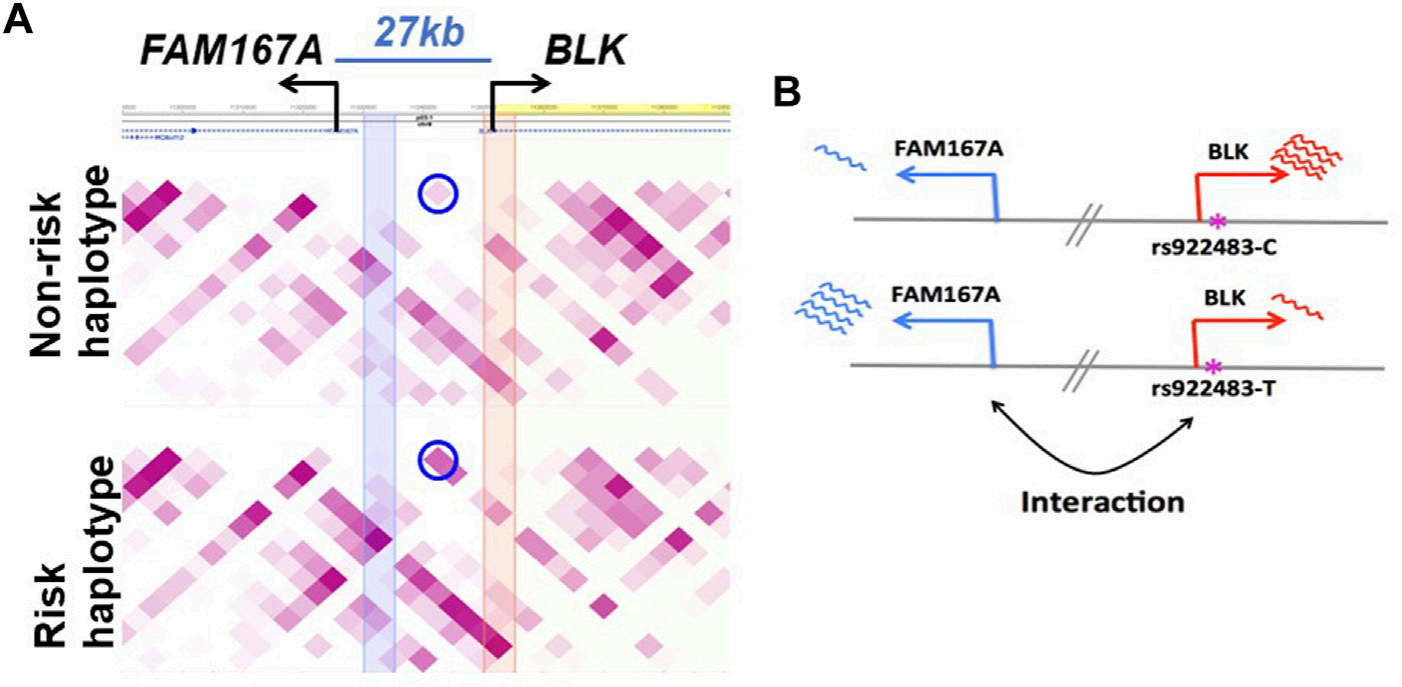
Haplotype-specific *FAM167A-BLK* promoter-promoter interactions in NA07000 cells. **(A)** *FAM167A*-*BLK* interaction is stronger on SLE risk haplotype. Shown is a heatmap view of Hi-C chromatin interaction frequencies on non-risk and risk haplotypes, darker purple representing higher interaction frequencies. Each square represents an interaction between two 5 kb genomic regions located at the diagonal extension intersecting the horizontal line. The circled squares represent interactions between two 5 kb regions located where blue and orange vertical bands meet the horizontal line (at *FAM167A* and *BLK* promoters). **(B)** *BLK* and *FAM167A* have anti-correlated expression patterns. *BLK* is less and *FAM167A* is more expressed on the risk (lower panel) than on the non-risk (upper panel) haplotype. Shown also is a putatively SLE causal promoter SNP rs922483 with its non-risk (C) and risk (T) alleles.

Function of the *FAM167A* gene located 27 kb upstream of *BLK* has recently been shown to be an activator of the non-canonical activation pathway of NFκB, which is potentially important for mechanisms of the inflammatory phenotypes with genetic associations at this locus (Mentlein et al., 2018; Yang et al., 2022). We, therefore, retain *FAM167A* as an SLE candidate gene, acknowledging the existing circumstantial evidence supporting *BLK* as a participant in the mechanism altering disease risk. *FAM167A* is upregulated 8-fold upon B cell receptor stimulation (publicly available RNA-seq data in GEO GSE61608). Further, RA-associated variants exhibit high LD (*r* ^2^ > 0.8) with a B cell selective cis-eQTL for *FAM167A* expression identified in a cohort of early RA patients (Thalayasingam et al., 2018). The haplotype-specific expression patterns of these two genes are anti-correlated: *BLK* is less expressed on the SLE risk haplotype than on the non-risk haplotype, whereas *FAM167A* is more expressed on the risk haplotype than on the non-risk haplotype (Figure 3B) (Hom et al., 2008). Our capture Hi-C data revealed that *FAM167A* and *BLK* promoters interact, with the interaction being stronger on risk-haplotype in NA07000, but not in NA07056 cells (Figures 2,3A). Our model is summarized in Figure 3B. It has previously been proposed that promoters can regulate other promoters *via* chromatin looping interaction (Xu et al., 2011). Many promoter-promoter chromatin interactions have previously been identified genome-wide (Chepelev et al., 2012; Li et al., 2012). Promoters with enhancer activity, the so-called enhancer-like promoters, have been described recently (Dao et al., 2017; Dao and Spicuglia, 2018). That the promoters of *FAM167A* and *BLK* genes may functionally interact in this manner is consistent with these data and remains an intriguing possibility.

Since SLE causal variants at *FAM167A-BLK* are very likely to be in the *BLK* promoter region (see discussion in Introduction) and given our findings above, we hypothesized that these variants may have functional effects on modulating *FAM167A* expression, and on disease risk, via haplotype-specific chromatin interaction between promoters of *BLK* and *FAM167A*. We thus sought to perturb *BLK* expression and measure changes in *FAM167A* expression.

**FIGURE 4:**
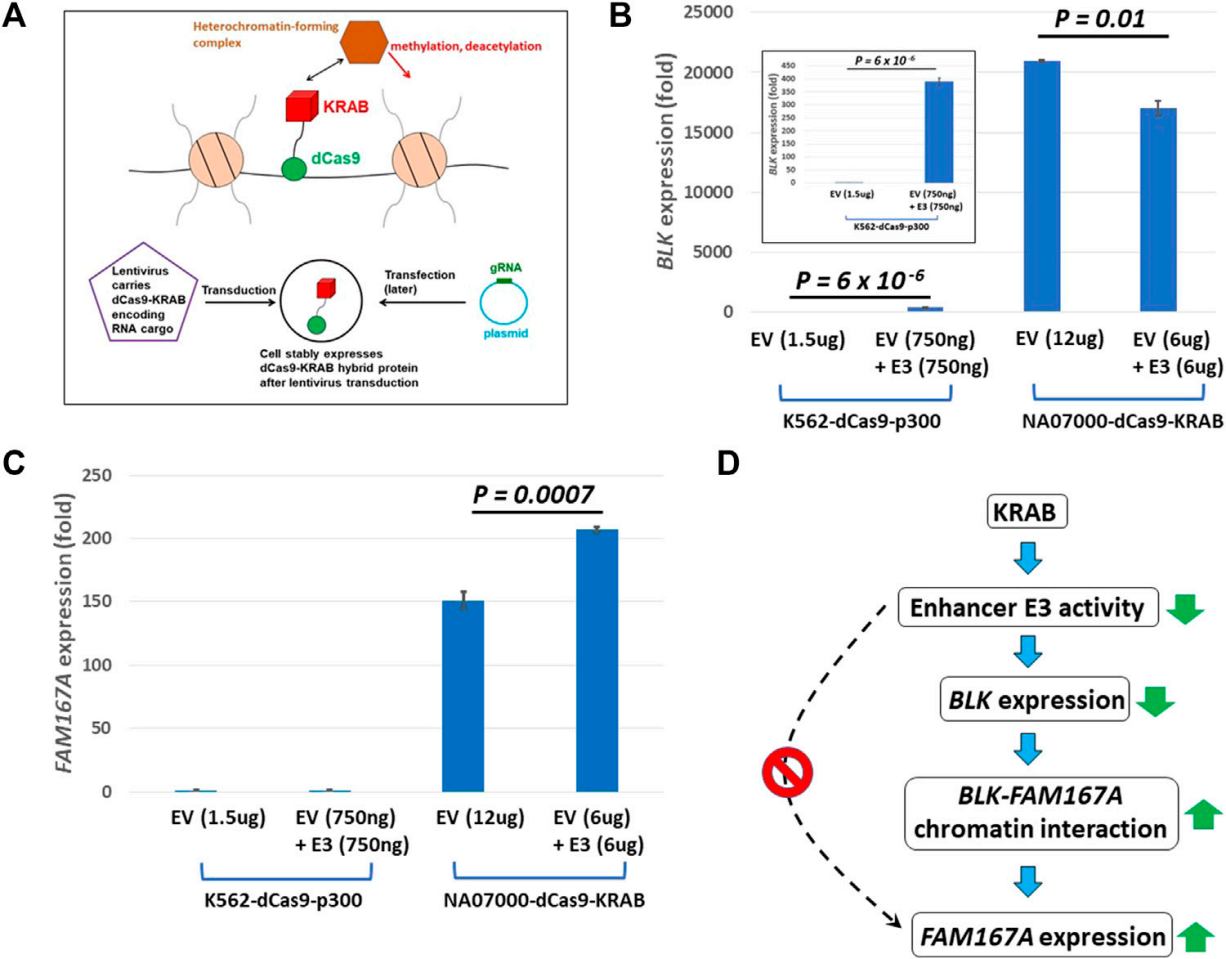
dCas9-KRAB targeting reveals regulation of *FAM167A* expression by *BLK* promoter region. **(A)** Schematics of epigenome editing (repressor KRAB) experiment in NA07000 LCL cell line. **(B)** Targeting of dCas9-KRAB to enhancer E3 in NA07000 cells results in down-regulation of *BLK*. Targeting of dCas9-p300 to enhancer E3 in K562 cells results in ∼400 folds up-regulation of *BLK* (two left-most bars in the main figure and the inset for an expanded view). For the schematics of dCas9-p300 experiments, see Figure 6A. **(C)** Targeting of dCas9-KRAB to enhancer E3 in NA07000 cells results in up-regulation of *FAM167A* expression. **(D)** A model to explain data in **(B–C)**. The dotted arrow with the red ‘STOP’ sign symbolizes the following finding from **(C)** (two left-most small bars grouped as ‘K562-dCas9-p300’ cell line): targeting of activator p300 to enhancer E3 in K562 cells did not result in up-regulation of *FAM167A* expression. This suggests that E3 does not directly regulate *FAM167A* expression. Rather, the effect of repressing E3 on *FAM167A* expression is mediated by *FAM167A-BLK* promoter-promoter interaction (see Figures 2,3). In **(B,C)**, the *BLK* and *FAM167A* expression values shown are relative to their expression levels in K562-dCas9-p300 cells transfected with empty vector (EV) (the left-most bars in **(B)** and **(C)** have values identically equal to 1). EV, empty vector.

We infected NA07000 LCL cells, in which *BLK* and *FAM167A* are expressed, with a lentivirus to express a dCas9-KRAB fusion protein for targeted gene repression (see Figure 4A) (Thakore et al., 2015). When localized to genomic DNA, KRAB recruits a heterochromatin-forming complex that causes histone methylation and deacetylation. We targeted dCas9-KRAB to the E3 enhancer region in NA07000 cells by transfection of plasmids expressing appropriate guide RNAs (gRNAs) (Supplementary Tables S3,S4). The *BLK* expression was down-regulated by ∼19% and *FAM167A* expression was up-regulated by ∼37% as a consequence of the targeting of dCas9-KRAB to the E3 enhancer region (Figures 4B,C).

The up-regulation of *FAM167A* may be a direct or an indirect consequence of perturbation of chromatin state at enhancer E3 by KRAB. In order to distinguish between these two possibilities, we chose K562 cells to perform epigenome editing to activate enhancer E3. The *BLK* and *FAM167A* genes are not expressed beyond the basal level in K562 cells and the *BLK* enhancers are not active since they have no H3K27ac signal in K562 cells (Supplementary Figure S1). We infected K562 cells with a lentivirus to express a dCas9-p300 fusion protein (Hilton et al., 2015; Klann et al., 2017) (see Figure 6A). The p300 is a histone acetyltransferase that acetylates H3K27. While *BLK* is strongly up-regulated by targeting of dCas9-p300 to enhancer E3 in K562 (Figure 4B), *FAM167A* expression did not change (Figure 4C). This suggests that the E3 region is not directly involved in regulating *FAM167A* expression. More likely, the effect of targeting dCas9-KRAB to E3 on *FAM167A* expression is indirect and is mediated by increased long-range chromatin interaction frequency between promoters of *BLK* and *FAM167A*, brought about by down-regulation of *BLK* expression (see the model in Figure 4D). We hypothesize that upon down-regulation of *BLK* transcription, an enhancer-like activity of *BLK* promoter goes up, which leads to its long-range chromatin interaction with *FAM167A* promoter and up-regulation of *FAM167A* expression.

### Haplotype-specific enhancer activity

**FIGURE 5:**
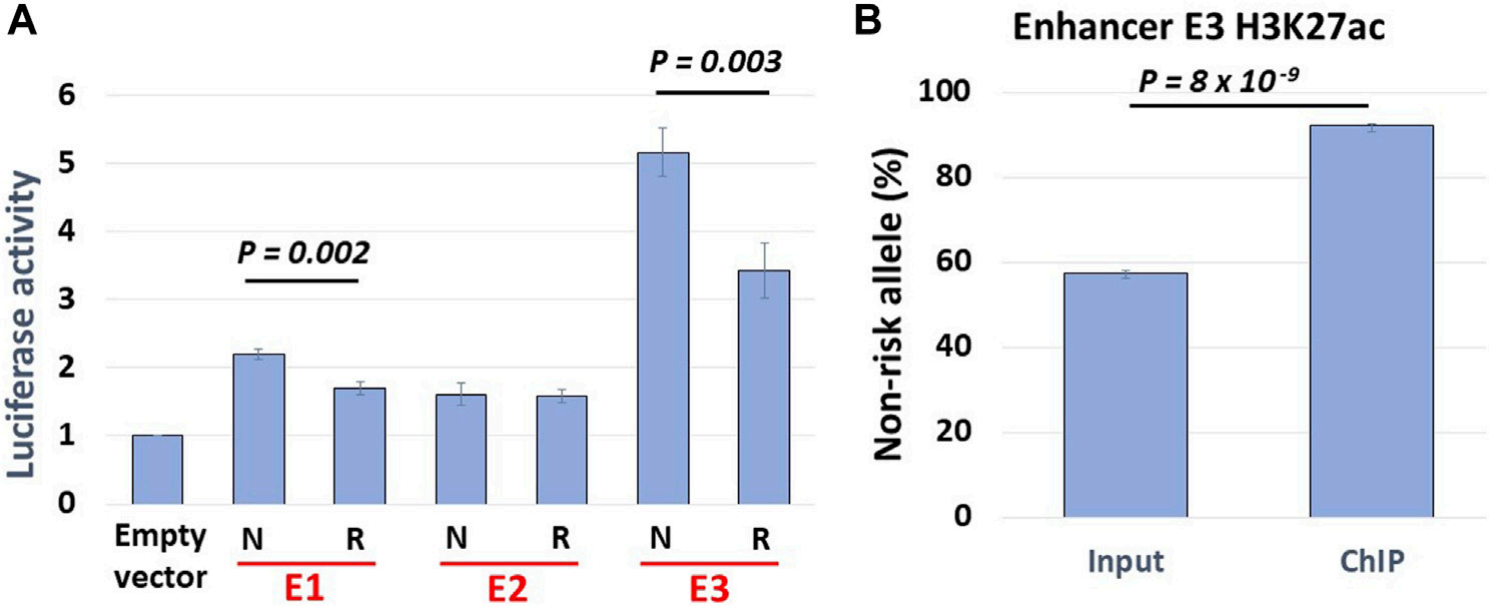
Haplotype-specific enhancer activity. **(A)** Allelic luciferase activities of enhancers in NA07056 cells. N: SLE non-risk haplotype sequence, R: SLE risk haplotype sequence. Non-risk enhancer E3 sequence reporter activity is ∼1.5x higher than that of risk sequence. **(B)** Allele-specific ChIP-qPCR at E3 enhancer in NA07056 cells in which the SNP rs2244931 is heterozygous. Non-risk to risk enhancer activity ratio in native chromatin context is significantly higher ∼8.5x (calculated as normalized ratio (N/R)_ChIP_/(N/R)_Input_; See Materials and Methods for details), presumably due to “amplification of E3 enhancer activity due to long-range chromatin interactions” effect (see haplotype-specific E1-E3 and E2-E3 interactions in Figure 2).

Our Hi-C data revealed haplotype-specific interactions of enhancers with the *BLK* promoter. We thus hypothesized that enhancer activities should be stronger on the SLE non-risk haplotype. We cloned NA07056 risk and non-risk haplotype enhancer sequences into luciferase reporter vectors and transfected LCLs with these constructs. The reporter assay data show that the activity of enhancer E3 is haplotype-specific, and is less active on the SLE risk haplotype (Figure 5A). This result is consistent with the chromatin interaction analysis above. Reporter experiments revealed that enhancer activity of non-risk E3 sequence is ∼1.5 times higher than the activity of risk E3 sequence (Figure 5A). Intriguingly, our allele-specific ChIP-qPCR experiments showed that the H3K27ac signal at E3 enhancer on non-risk haplotype is ∼8.5 times higher than on the risk haplotype (Figure 5B). We hypothesize that the allelic differences in regulatory element activities are determined not only by local DNA sequence variations at the element but also by allelic differences at distal sites, due to the amplification effect of chromatin looping interactions in the 3D chromatin context. We have tested our hypothesis for one enhancer (E3) in one cell line in our experiments, suspecting that this is a more general phenomenon and that analogous findings would hold true also at many other regulatory regions.

### Epigenome editing reveals participating elements in *BLK* regulation

**FIGURE 6:**
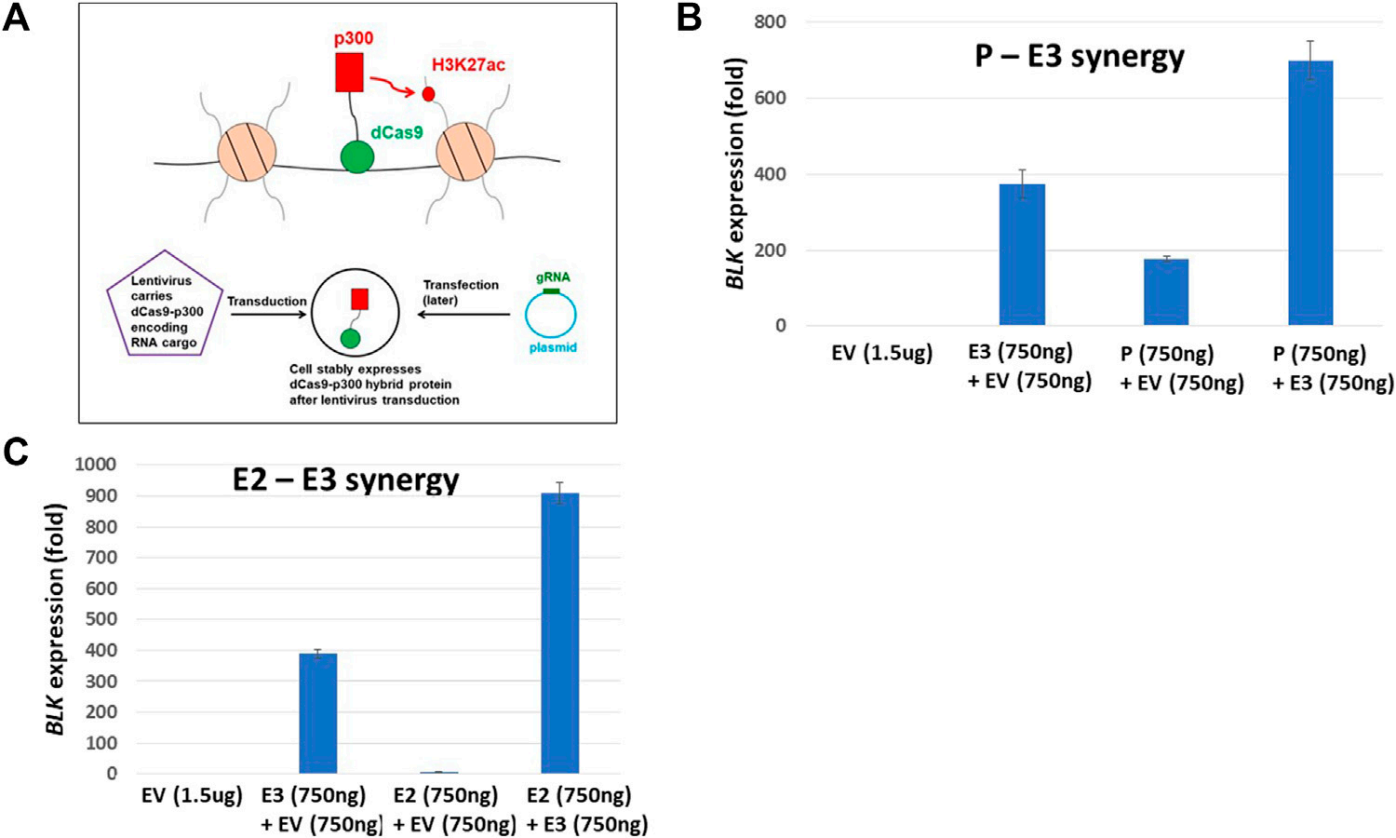
dCas9-p300 targeting reveals synergistic interaction of regulatory elements. **(A)** Schematics of epigenome editing (activator p300) experiment in K562 cell line. **(B)** P-E3 synergy: upregulation of *BLK* expression when both the *BLK* promoter P and the enhancer E3 are targeted by p300 protein is significantly higher than when p300 is targeted to either P (*p*-value = 0.004) or E3 (*p*-value = 0.004) alone. **(C)** E2-E3 synergy: upregulation of *BLK* expression when both enhancers E2 and E3 are targeted by p300 protein is significantly higher than when p300 is targeted to either E2 (*p*-value = 0.0008) or E3 (*p*-value = 0.0008) alone. In B-C, the *BLK* expression values shown are relative to the *BLK* expression level in K562-dCas9-p300 cells transfected with empty vector (EV) (the left-most bars in **(B)** and **(C)** have values identically equal to 1).

Our Hi-C data revealed haplotype-specific chromatin looping interactions between enhancers and the promoter at the *BLK* locus. Based on these findings, we hypothesize that enhancers and promoter pairs synergistically regulate *BLK* expression. For the lack of baseline *BLK* expression and the absence of an H3K27ac signal at the enhancers (Supplementary Figure S1), we again chose K562 cells, here performing epigenome editing to activate *BLK* expression and test the hypothesis. We infected K562 cells with a lentivirus to express a dCas9-p300 fusion protein (Hilton et al., 2015; Klann et al., 2017) (see Figure 6A). The p300 is a histone acetyltransferase that acetylates H3K27. We targeted dCas9-p300 to E2, E3 and promoter regions singly and in combinations in K562 cells by transfection of plasmids expressing appropriate guide RNAs (gRNAs) (Supplementary Tables S3,S4). Strong synergistic activation of the *BLK* gene (500 to 900-fold increases) was observed for E2-E3 and E3-promoter targeting by p300 (Figures 6B,C), thereby confirming the regulatory role of this genome region for *BLK*.

### Multiple genetic variants contribute to variance in *BLK* expression

Since gene regulation is a complex process involving interactions of multiple regulatory regions and protein complexes, we reasoned that many genetic variants may contribute to variance in *BLK* expression. We analyzed gene expression and genotype data from 344 European individuals (Lappalainen et al., 2013), henceforth denoted as dataset D344, using the genetic relationship matrix approach as implemented in GCTA software (Yang et al., 2010) to estimate *BLK* gene expression variance explained by various sets of SNPs. The SLE-associated proxy rs922483 alone explained around 25% of expression variance. Inclusion of all SNPs in putative regulatory regions of *BLK*, as defined by the H3K27ac epigenetic mark and REST binding region (see Figure 2; Supplementary Table S7), increased the variance explained to 44%, supporting the contention that multiple SNPs at the *BLK* locus explain variance in gene expression.

### Enhancer haplotypes modulate *BLK* expression

Given our findings, we sought to find more direct evidence for the causal effects of enhancer haplotypes on *BLK* expression. To this end, we derived an SLE credible set of 11 SNPs, termed BLK-CS-11, from existing genotype data (see Materials and Methods, and Supplementary Table S5) and used phased genotype data for individuals of European ancestry from the 1000 Genomes Project (1000genomes.org) to extract haplotype sequences of the *BLK* locus. We then computed frequencies of different promoter haplotype sequences in the dataset.

**FIGURE 7:**
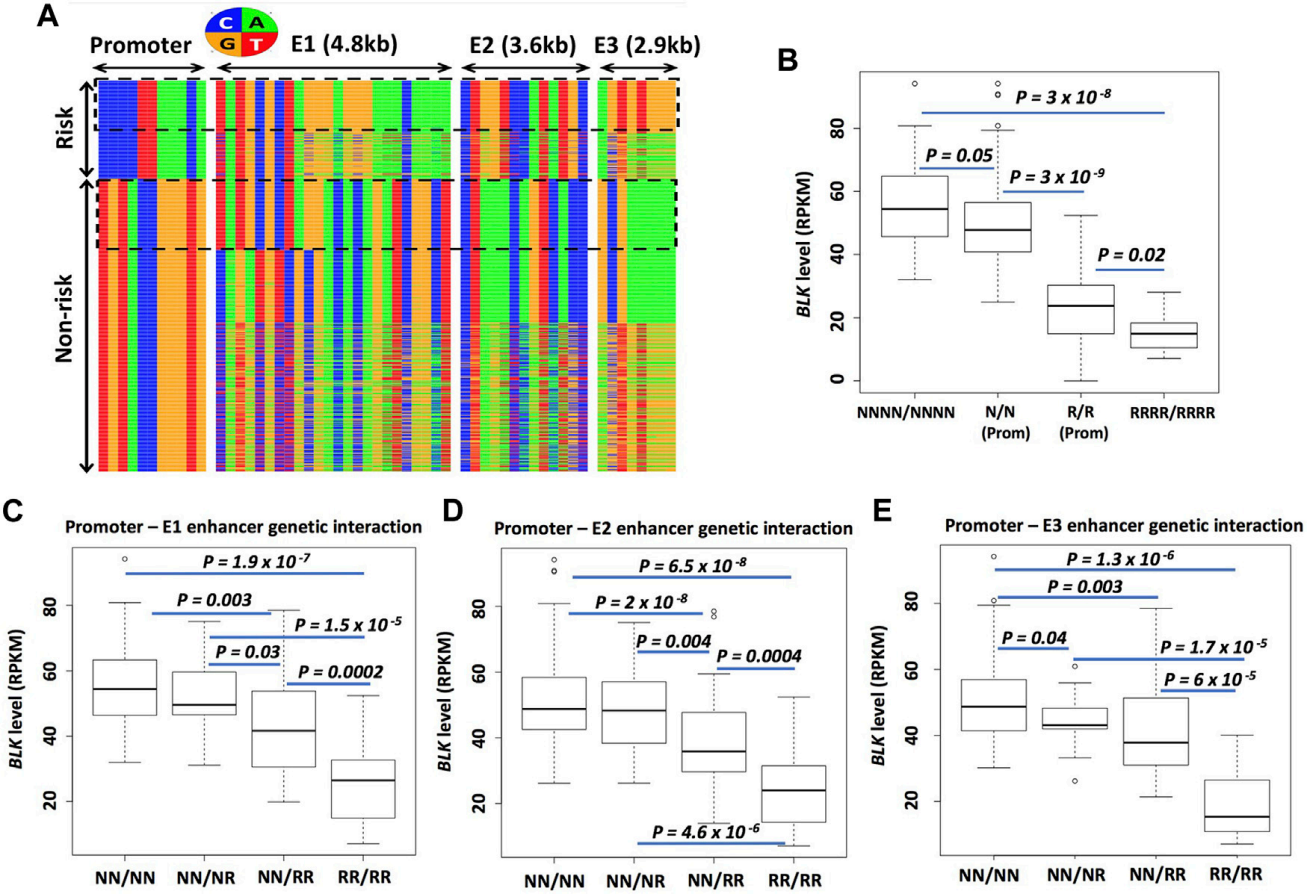
Enhancer haplotypes modulate *BLK* expression. **(A)** Promoter-E1-E2-E3 haplotypes conditioned on promoter risk and non-risk haplotypes. Each row represents a haplotype and each column - a SNP. Nucleotides are color-coded as shown in the circle above the haplotype heatmap **(B)** Risk haplotypes at enhancers lower *BLK* expression. **(C–E)** “Risk dosage” effect. The *BLK* expression level tends to decrease as the total number of ‘risk’ haplotypes in the regulatory regions (promoter and enhancers E1, E2, E3) increases. In **(B–E)**, N means non-risk haplotype and R means risk haplotype. NNNN/NNNN and RRRR/RRRR denote multilocus genotypes at P-E1-E2-E3; N/N and R/R denote multilocus genotypes at the promoter; NN/NN, NN/NR and so on in **(C–E)** denote multilocus genotypes at P-E1, P-E2 or P-E3. See Materials and Methods for details on the notation.

The frequency of promoter risk haplotype CCCCTTAAACA (based on 11 SNPs in BLK-CS-11) (Figure 7A), which we denote henceforth as prom-R, is 24%. The most frequent non-risk haplotype TGTACCGGGTG, which we denote as prom-N, is present at 72%. In subsequent analyses, we will focus on these two main haplotypes, which together account for 96% of all European haplotypes. The rows in the heatmap plot in Figure 7A correspond to the haplotypes, with the rows labeled as “Risk” representing promoter “risk” haplotypes and the rows labeled as ‘Non-risk’ representing promoter “non-risk” haplotypes, respectively.

We next asked, conditioned on the promoter haplotype, what are the local haplotype sequences at candidate enhancer E1, E2, and E3 regions defined by islands of H3K27ac enrichment? The promoter and enhancer haplotype sequences are displayed in Figure 7A. The haplotypes are grouped based on promoter haplotype: risk for prom-R (upper block) and non-risk for prom-N (lower block). . We conditioned on the promoter haplotype prom-R and counted different local haplotypes at E1, E2, and E3. The most frequent E1, E2, and E3 haplotypes, conditioned on prom-R haplotype, denoted as E1-R, E2-R, and E3-R, respectively, are present in 87%, 85%, and 54% of all prom-R haplotypes, respectively. Our choice of the label “R” in the naming of these enhancer haplotypesmay seem arbitrary; the use of this label emphasizes the effect of these haplotypes on *BLK* expression and disease risk discussed below. Interestingly, the haplotype combination (prom-R, E1-R, E2-R, E3-R), which can be seen in the upper dotted box in Figure 7A, is frequent and constitutes 48% of all promoter-E1-E2-E3 haplotypes conditioned on prom-R and 11% of all European subject chromosomes.

Similarly, the most frequent E1, E2, and E3 haplotypes, conditioned on prom-N haplotype, denoted as E1-N, E2-N, and E3-N, respectively, are present in 25%, 70%, and 50% of all prom-N haplotypes, respectively. Interestingly, the haplotype combination (prom-N, E1-N, E2-N, E3-N), which can be seen in the lower dotted box in Figure 7A, is frequent and constitutes 24% of all promoter-E1-E2-E3 haplotypes conditioned on prom-N. All other alternative local haplotypes at E1, E2, and E3, different from N and R haplotypes defined above, are denoted by E1-A, E2-A, and E3-A, respectively. We will use simplified notation, such as RNRN for (prom-R, E1-N, E2-R, E3-N) haplotype, subsequently.

We next asked: how does the *BLK* mRNA level depend on different haplotype combinations at the promoter, E1, E2, and E3? To answer, we used RNA-seq expression data from dataset D344 [gene expression from 344 European individuals (Lappalainen et al., 2013)]. We have defined two groups of samples based on their multilocus genotypes (i.e., pairs of haplotypes in each individual) NNNN/NNNN and RRRR/RRRR. We have also defined two more groups of samples based only on promoter multilocus genotypes prom-N/prom-N and prom-R/prom-R, denoted N/N and R/R, respectively. The boxplot of expression levels in these 4 groups is shown in Figure 7B. There is a clear “risk-dosage” dependent decrease in *BLK* expression level in these data, with the contributions of “risk” from both the promoter and enhancer variants. Analyses for *BLK* promoter-enhancer pairs, shown in Figures 7C–E, support a risk-dosage model according to which the risk status of local haplotypes at cis-regulatory regions cumulatively contributes to a reduction in *BLK* expression. As defined earlier, alternative local haplotypes at E1, E2, and E3, different from N and R haplotypes, are denoted as “A”. By replacing N’s with A’s in the analyses, we found almost identical results as above, suggesting that A and N enhancer haplotypes have similar effects on *BLK* expression (data not shown).

### Genetic interactions at *FAM167A-BLK* locus are enriched in regulatory regions

We have provided evidence supporting the hypothesis that enhancer haplotypes can influence *BLK* expression by acting as genetic modifiers of promoter haplotypes. However, we excluded from our analysis genetic variants outside of gene regulatory elements/regions (GREs). What is the contribution of the non-GRE variants to *BLK* expression and do these variants interact? To address this question, we analyzed data D344 [gene expression data from 344 European individuals (Lappalainen et al., 2013)]. For each pair of SNPs, we fitted a linear (y = b_0_ + b_1_x_1_ + b_2_x_2_) and an interaction (y = b_0_ + b_1_x_1_ + b_2_x_2_ + b_12_x_1_x_2_) model to *BLK* expression and genotype data (y denotes expression level and x_1_, x_2_ denote 0/1/2 coded genotypes of the two SNPs under consideration). The log-likelihood ratio test was used to determine statistically significant genetic interactions. If our hypothesis is true, then the genetic interactions are predicted to be predominantly between SNPs located at GREs. However, SNPs at the *BLK* locus are in high linkage disequilibrium with each other; therefore, the statistically identified genetic interactions may not necessarily be between genetic variants that causally influence *BLK* expression.

**FIGURE 8:**
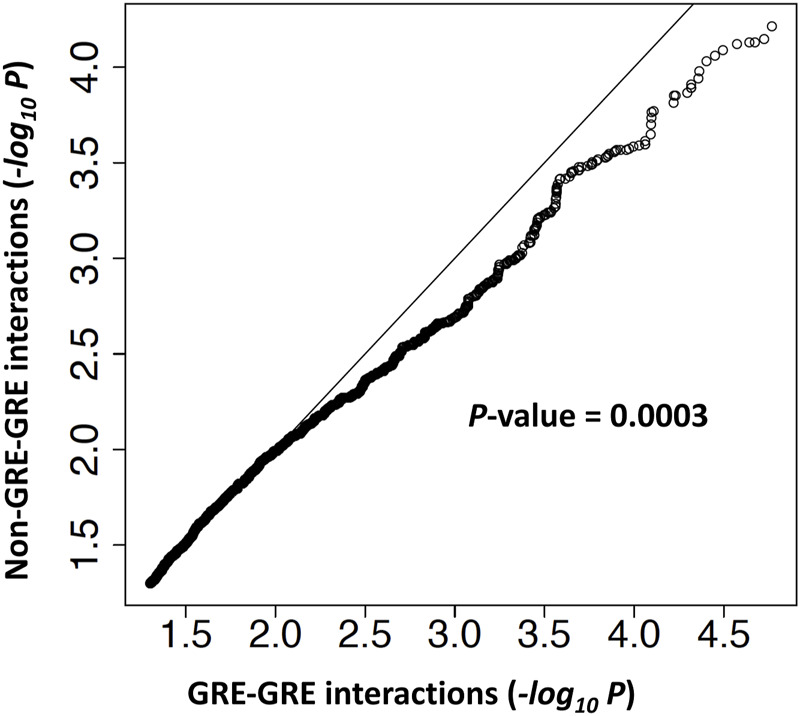
Genetic interactions are enriched at gene regulatory elements. A quantile-quantile (Q–Q) plot of -log10 *p*-values from the log-likelihood ratio test of genetic interaction. Genetic interactions were separated into two groups. In the GRE-GRE group, both SNPs are located in gene regulatory regions. In the non-GRE-GRE group, at least one SNP is located outside of the regulatory regions.

In order to perform an unbiased test of genetic interaction enrichment at regulatory regions, we separated genetically interacting SNP pairs identified by our statistical analysis into two groups: 1) SNPs pairs that are in two different gene regulatory regions (GRE-GRE group) and 2) SNP pairs with at least one SNP not in a regulatory region (non-GRE-GRE group). The genomic coordinates of gene regulatory regions and details of the analysis are given in Materials and Methods. A ranked list of–log_10_(*p*-value) of genetic interactions in each group was generated. Quantile-quantile analysis revealed that genetic interactions in the GRE-GRE group are more significant than in the non-GRE-GRE group (*p*-value = 0.0003) (Figure 8), thereby demonstrating enrichment of genetic interactions between gene regulatory elements.

## Materials and methods

### Cell culture

K562 cells were obtained from the American Tissue Collection Center (ATCC). NA07056 and NA07000 LCLs were obtained from the Coriell Institute for Medical Research cell repository. K562 cells were maintained in Iscove’s Modified Dulbecco’s Medium supplemented with 10% FBS and 1% penicillin-streptomycin. LCLs were maintained in RPMI medium supplemented with 10% FBS and 1% penicillin-streptomycin. K562 and LCLs were grown at 37°C in 5% CO_2_.

### Capture Hi-C experiments

Capture Hi-C libraries were prepared according to the protocol described in (Jäger et al., 2015). The protocol consists of two parts: Hi-C library preparation and target enrichment (Figure 1A). A SureSelect Custom Target Enrichment Library covering a 3 Mb region in the 8p23.1 (hg19 coordinates: chr8:8,190,000–11,838,000) was designed using eArray software (Agilent). Hi-C library preparation, comprising chromatin fixation, HindIII digestion, biotin labelling, ligation, and crosslink reversal was performed as described in (Rao et al., 2014) with minor modifications described in (Jäger et al., 2015). Target enrichment was performed according to the SureSelect protocol (Agilent) with minor modifications described in (Jäger et al., 2015). We have prepared 10 capture Hi-C libraries from 5 independent batches of NA07000 cells and 4 capture Hi-C libraries from 2 independent batches of NA07056 cells. The libraries were sequenced on Illumina HiSeq 2,500 system, producing 461 million and 206 million paired-end 2 × 125 bp reads for NA07000 and NA07056, respectively.

### Allele-specific luciferase assay

We amplified ∼2 kb DNA fragments located in the E1, E2, and E3 enhancer regions (see Figure 2; the hg19 coordinates of the fragments are chr8:1,1,391,971–11,391,971, chr8:11,394,297–11,396,127 and chr8:11,402,768–11,404,795, respectively) from the genomic DNA of NA07056 lymphoblastoid cells using PCR with primers listed in Supplementary Table S6. The PCR products were cloned into pCR2.1-TOPO vector (catalog #K4500-01, Invitrogen) and sub-cloned into pGL4 luciferase reporter vectors (catalog #E6651, Promega). The bacterial cells were then transformed, and single-cell colonies were isolated and Sanger-sequenced to identify SLE risk and non-risk clones using phased haplotype data from the 1000 Genomes Project.

An internal control reporter vector containing Renilla luciferase was simultaneously transfected with our experimental vectors as a control for assay-to-assay variability. One microgram of each vector was transfected into the NA07056 (10^6^ cells per sample in triplicate). Cells were then incubated at 37°C for 24 h. Luciferase activity was measured with the Dual-Luciferase Reporter Assay System (catalog #E1960, Promega). Luciferase activity was normalized through the division of *BLK* risk or non-risk construct reporter activity by the reporter activity of the pRL-TK-Renilla luciferase construct. The mean and standard error of measurement were calculated on the basis of the normalized luciferase activities. The one-sided Student’s t-test was used to compare the N and R groups in Figure 5A.

### Allele-specific H3K27ac ChIP-qPCR

Three independent chromatin immunoprecipitation (ChIP) experiments in NA07056 cells were carried out using antibodies against H3K27ac (catalog #C15410196, pAb-196–050, Diagenode) following a standard protocol. Enhancer E3 (see Figure 2) SNP rs2244931 is heterozygous in NA07056 (C/G, where G is on the SLE risk haplotype). Allelic H3K27ac levels at rs2244931 were quantified using TaqMan custom SNP Genotyping Assay, TaqMan Genotyping Master Mix (catalog # 4,371,353, Applied Biosystems), and input/ChIP DNA. The allelic ratios in the ChIP and input DNA were determined by fitting log2 transformed VIC/FAM ratios to a standard curve constructed from DNA with known rs2244931 allelic ratios obtained by mixing gRNA from cell lines homozygous for rs2244931 (C/C and G/G) as well as heterozygous (C/G). The normalized non-risk to risk allele ratio of the H3K27ac signal was determined as the ratio (N/R)_ChIP_/(N/R)_Input_ of non-risk/risk allele ratios in ChIP and input DNA samples. In Figure 5B, the percentage of the non-risk allele RT-qPCR signal in input DNA is ∼57.5% and that of the risk allele is ∼42.5%. Similarly, the non-risk allele RT-qPCR signal in ChIP DNA constitutes ∼92% and the risk allele signal constitutes ∼8%. Thus, the normalized (true) non-risk to risk ratio of the H3K27ac signal is (92/8)/(57.5/42.5) = 8.5. The one-sided Student’s t-test was used to compare percentages of non-risk alleles RT-qPCR signals in input and ChIP DNA (Figure 5B).

### CRISPR epigenome editing

The plasmid pLV-dCas9-p300-P2A-PuroR was a gift from Charles Gersbach (Addgene plasmid # 83,889) (Klann et al., 2017). The plasmid pLV hU6-sgRNA hUbC-dCas9-KRAB-T2a-GFP was a gift from Charles Gersbach (Addgene plasmid # 71,237) (Thakore et al., 2015). The plasmid pSPgRNA was a gift from Charles Gersbach (Addgene plasmid #47108) (Perez-Pinera et al., 2013). For dCas9-p300 and dCas9-KRAB experiments, lentivirus was produced by transfecting HEK293 cells with pLV-dCas9-p300-P2A-PuroR and pLV hU6-sgRNA hUbC-dCas9-KRAB-T2a-GFP plasmids, respectively, at Cincinnati Children’s Hospital Medical Center’s Viral Vector Core facility.

For dCas9-p300 experiments, 5 million K562 cells were incubated for 3 days with the concentrated dCas9-p300 lentivirus at the cell to virus ratio of 1:5 in the presence of 8ug/ml polybrene. After 3 days, virus-infected cells were selected with 2 ug/ml of puromycin. We named the resulting cells K562-dCas9-p300.

For dCas9-KRAB experiments, 10 million NA07000 cells were spinfected (in 12 well plate, 1 million cells/ml of media, centrifuged at 800Xg for 2 h at 30°C) with the concentrated dCas9-KRAB lentivirus at the cell to virus ratio of 1:5 in the presence of 8 ug/ml polybrene. After spinfection, 1 ml of fresh media was added to each well (2 ml total) and cultured for 4 days at 37°C. Cells were then washed 3 times with fresh cultured media and continued to grow for 10 more days. After 2 weeks of infection, GFP + virus-infected cells were sorted and cultured to grow more GFP + virus-infected cells. We named the resulting cells NA07000-dCas9-KRAB.

For the regions P, E2 and E3 in Figure 2, we designed several gRNAs targeting these regions (see Supplementary Table S3 for the list of gRNA oligos and Supplementary Table S4 for Addgene IDs of the plasmids generated in this study). Each gRNA oligo pair was phosphorylated using T4 PNK, annealed and cloned into BbsI-digested pSPgRNA plasmid. For each of the P, E2, and E3 regions, we generated equimolar pools of gRNA plasmids (see Supplementary Tables S3,S4).

For *BLK* repression experiments, NA07000-dCas9-KRAB cells were transfected with E3 gRNA plasmid together with the empty vector (EV + E3) or the empty vector alone (EV) (see Figure 4). The empty vector is simply the intact pSPgRNA plasmid. As indicated in Figure 4, the total amount of plasmids used in each transfection was 12 ug, with 6 ug of EV plasmid included whenever it was necessary in order to have a balanced total DNA amount of 12 ug. The optimal amount, 12 ug, of plasmids used for transfection in NA07000-dCas9-KRAB experiments above was determined from exploratory transfection experiments performed with E3 gRNA plasmid alone at varying DNA amounts and selecting the amount which resulted in the largest down-regulation of *BLK* expression (data not shown).

For synergy experiments, K562-dCas9-p300 cells were transfected with pairs of gRNA pools (P + E3 or E2+E3), a single gRNA pool together with the empty vector (EV + P, EV + E2, or EV + E3) or the empty vector alone (EV) (see Figure 6). The empty vector is simply the intact pSPgRNA plasmid. As indicated in Figure 6, the total amount of plasmids used in each transfection was 1.5 ug, with 750 ng of EV plasmid included whenever it was necessary in order to have a balanced total DNA amount of 1.5 ug. The optimal amount, 1.5 ug, of plasmids used for transfection in K562-dCas9-p300 experiments above was determined from exploratory transfection experiments performed with E3 gRNA plasmid alone at varying DNA amounts and selecting the amount which resulted in the highest upregulation of *BLK* expression (data not shown).

At 24 h, cells were harvested and mRNA was extracted using Dynabeads mRNA DIRECT kit (catalog # 61,006, Invitrogen). cDNA was generated using SuperScript™ IV VILO™ Master Mix kit (catalog # 11,756,050, Invitrogen). The *BLK* and *FAM167A* mRNA levels were measured by RT-qPCR using TaqMan Fast Advanced Master mix (catalog# 4,444,963, ThermoFisher Scientific) and TaqMan probes for *BLK* (Hs01017458_m1), *FAM167A* (Hs00697562_m1) and *GAPDH* (Hs03929097_g1). The one-sided Student’s t-test was used to compare expression levels in Figures 4B,C and Figures 6B,C.

### Analysis of capture Hi-C data

The capture Hi-C paired-end sequencing reads were mapped to the SNP-masked hg19 human genome using HiC-Pro software as follows (Servant et al., 2015) (Figure 1B). First, we obtained phased genotype VCF files for NA07000 and NA07056 cell lines from the 1000 Genomes Project (version v5a.20,130,502), and generated two SNP-masked hg19 genomes files for these cell lines. The heterozygous (Ref/Alt) and homozygous alternative allele (Alt/Alt) loci in the genomes were masked as “N” in order to mitigate the reference mapping bias. The individual reads in each mapped paired-end read are evaluated for the presence of heterozygous SNPs and labeled as one of the following using phased genotype data from the 1000 Genomes Project: parent-1 allele (M), parent-2 allele (P), or allele unassigned (UA). The M-M, M-UA, and UA-M paired-end reads were grouped as “parent-1” and P-P, P-UA and UA-P paired-end reads were grouped as “parent-2” to compute chromatin interaction frequencies on two parental chromosomes at 5 kb resolution. To identify differences in chromatin interaction frequencies of homologous chromosomes, we determined haplotype-specific interaction frequency matrices in 5 kb bins for each replicate library. For n replicate libraries from each cell line, we computed n matrices for parent-1 haplotype and n matrices for parent-2 haplotype. Treating the entries of these matrices as sequence count data with the study design {M, M, … , M, P, P, … , P} (n consecutive M’s followed by n consecutive P’s), we determined differences between chromatin interaction frequencies on parent-1 and parent-2 chromosomes using multiHiCcompare (version 1.10.0) method (Stansfield et al., 2019) with the following parameters: make_hicexp (zero.*p* = 0.8, A.min = 5), cyclic_loess (span = 0.2), logfc_cutoff = 0.5, logcpm_cutoff = 0.5, p. method = “fdr” and p.adj_cutoff = 0.05. Differential chromatin interaction data was uploaded as custom tracks to WashU Epigenome Browser (https://epigenomegateway.wustl.edu/) and the images were then exported (Figures 2,3A).

### Enhancer haplotype analysis

Using Bayesian approaches to describe “credible sets” of disease-causal SNPs following published methods (Wellcome Trust Case Control Consortium et al., 2012), we have identified the smallest set of SNPs accounting for 95% of the posterior probability from 3,892 European-American SLE cases and 3,464 controls (Rasmussen et al., 2011). This credible causal set at *FAM167A-BLK* locus, henceforth denoted as BLK-CS-12, consists of 12 SNPs that are in high linkage disequilibrium (LD) (*r* ^2^ > 0.9), spans a 13 kb region near *BLK* promoter. Conditional analysis on any of the 12 markers reduces association for the other 11, consistent with a single genetic association. Since the 12 markers are in perfect linkage disequilibrium with each other, without loss of generality, we have omitted a rare indel variant (rs202125301) from BLK-CS-12 and used the set of 11 SNPs (Supplementary Table S5), which we denote as BLK-CS-11, in our analyses leading to Figure 7.

We have written a collection of Perl and R codes to perform analyses leading to Figure 7. We obtained phased genotype VCF file for the European population (GBR, FIN, CEU, IBS, and TSI; 503 individuals in total) from the 1000 Genomes Project (version v5a.20,130,502), and retained genetic variants with minor allele frequency (MAF) ≥ 5% for downstream analysis. From the resulting VCF file, we have extracted phased genotype data for 11 SNPs from the BLK-CS-11 set, 24 SNPs from the enhancer E1 region (hg19 coordinate chr8:11,387,781–11,392,710), 13 SNPs from enhancer E2 region (chr8:11,393,832–11,397,791) and 8 SNPs from enhancer E3 region (chr8:11,402,260–11,405,263). These SNP numbers correspond to the number of columns (11 + 24+13 + 8) in the heatmap plot in Figure 7A. From the phased genotype data for 11 promoter variants from BLK-CS-11, we extracted 1,006 haplotypes (from 503 individuals), of which 725 are “non-risk” (TGTACCGGGTG), 243 are “risk” (CCCCTTAAACA) and 38 are “idiosyncratic.” We omitted the idiosyncratic haplotypes from the downstream analyses. The rows in the heatmap plot in Figure 7A correspond to the haplotypes, with the rows labeled as ‘Risk’ representing 243 promoter “risk” haplotypes and the rows labeled as “Non-risk” representing 725 promoter “non-risk” haplotypes, respectively. Each entire row in the heatmap represents the concatenation of promoter, E1, E2, and E3 local haplotypes from the same chromosome.

Conditioned on the promoter haplotype, we further stratified haplotypes on the basis of the local haplotype sequences at candidate enhancer E1, E2, and E3 regions as described in detail in the Main text. In Figure 7B, NNNN/NNNN denotes a multilocus genotype at P-E1-E2-E3. It denotes all individuals in the dataset D344 [gene expression and genotype data from 344 European individuals (Lappalainen et al., 2013)] who possess the P-E1-E2-E3 haplotype shown in the lower dotted box in the ‘Non-risk’ block in Figure 7A on both parental chromosomes 8. Similarly, RRRR/RRRR denotes two identical haplotypes from the upper dotted box in the “Risk” block in Figure 7A.

N/N (prom) in Figure 7A denotes individuals from the D344 dataset who possess prom-N (i.e. non-risk) promoter haplotype (TGTACCGGGTG) on both chromosomes 8, with the local haplotypes at E1, E2, and E3 unspecified. In other words, an individual from the N/N (prom) group can have any promoter-E1-E2-E3 haplotype shown in the “Non-risk” block in Figure 7A. Similarly, an individual from the R/R (prom) group can have any promoter-E1-E2-E3 haplotype shown in the “Risk” block in Figure 7A.

NN/RR in Figure 7E denotes the multilocus genotype (TGT​ACC​GGG​TG)-(GCG​AAA​AA)/(CCC​CTT​AAA​CA)-(AGT​GTG​GG) at promoter-E3, with the local haplotypes at E1 and E2 unspecified. Other multilocus genotypes in Figures 7C–E are similarly defined.

The one-sided Student’s t-test was used to compare *BLK* expression level distributions in the two groups of individuals in Figures 7B–E.

### Statistical test of genetic interactions enrichment in regulatory regions

From the dataset D344, we retrieved SNPs from the 143 kb region R143k (hg19 coordinate chr8:11, 331, 000–11,474,000) at the *BLK* locus. The log-likelihood ratio test was used to determine statistically significant genetic interactions. For each pair of SNPs from the region R143k, we fitted a linear (y = b_0_ + b_1_x_1_ + b_2_x_2_) and an interaction (y = b_0_ + b_1_x_1_ + b_2_x_2_ + b_12_x_1_x_2_) model to *BLK* expression and genotype data, where y denotes expression level and x_1_, x_2_ denote 0/1/2 coded genotypes of the two SNPs under consideration. The log-likelihood ratio test was used to determine statistically significant genetic interactions i.e. the test of whether the interaction model explains the observed data better than the linear model does.

For each pair of SNPs from the region R143k, we thus have a *p*-value from the log-likelihood test of genetic interaction. To test the hypothesis that genetic interactions are enriched in gene regulatory regions at the *BLK* locus, we separated SNPs from the R143k region into two groups: SNPs from the putative regulatory regions of *BLK*, as defined by the H3K27ac epigenetic mark and REST binding region (see Figure 2; Supplementary Table S7) (denoted as GRR) and SNPs outside of the putative regulatory regions. For each SNP pair (SNP1, SNP2), if both SNPs belong to GRR, then the SNP pair is of the “GRE-GRE” type in the notation of Figure 8. If at least one of the two SNPs is outside of GRR, the SNP pair is of the “Non-GRE-GRE” type. The question of whether the genetic interactions are predominantly between SNPs located in gene regulatory regions thus reduces to the problem of comparison of the distribution of the genetic interaction log-likelihood *p*-values defined above for the group of GRE-GRE SNP pairs and non-GRE-GRE SNP pairs. We used the quantile-quantile plot (Q-Q plot) plot to show that the *p*-values of SNP-SNP interactions in the GRE-GRE group are more significant than those in the non-GRE-GRE group. One-sided Kolmogorov-Smirnov test was used to compare the distribution of the log-likelihood *p*-values to obtain *p*-value = 0.0003 shown in Figure 8.

## Discussion

Why are our findings on genetic interactions important for SLE? After all, credible causal SNPs are clearly localized to the *BLK* promoter region. Our hypothesis is that SNPs located in enhancer regions can further contribute to disease risk by modulating the effects of promoter SNPs on *BLK* expression. Indeed, a previous model proposes that genetic variants in weak linkage disequilibrium (LD) with risk variants can influence disease risk via physical interactions in the 3D chromatin context (Corradin et al., 2016). Our functional experiments and genetic analyses provide explicit evidence supporting the validity of this model.

In this study, we presented evidence that global haplotype-specific 3D chromatin interactions between regulatory regions can have a strong influence on local allelic gene expression, and consequently, on disease risk. Our capture Hi-C data revealed that many long-range chromatin interactions in the 8p23.1 region are haplotype-specific. Focusing specifically on the *FAM167A-BLK* locus, we found that the *BLK* promoter and the enhancer E3 located 52 kb downstream from the promoter haplotype-specifically interact in the 3D chromatin context, with the interaction being weaker on the SLE risk haplotype, consistent with the reduced expression of *BLK* on the risk haplotype (Figures 2,3). Interestingly, we found that the enhancer E3 interacts with haplotype-specificity with two nearby enhancers E1 and E2, which are members of the same super-enhancer cluster to which E3 belongs (Hnisz et al., 2013), with the interactions being weaker on the risk haplotype. We hypothesized that these interactions amplify the enhancer activity of E3. To test this hypothesis, we have performed allele-specific enhancer reporter assay and allele-specific H3K27ac ChIP-qPCR experiments (Figure 5). Our reporter experiments revealed that enhancer activity of non-risk E3 sequence is ∼1.5 times higher than the activity of risk E3 sequence (Figure 5A). Consistent with our ‘local enhancer activity amplification due to long-range chromatin interactions’ hypothesis, allele-specific ChIP-qPCR experiments demonstrated that H3K27ac signal at E3 enhancer on non-risk haplotype is ∼8.5 times higher than on risk haplotype (Figure 5B), the reasoning being that enhancer activity in reporter assays lacks long-range chromatin context whereas the H3K27ac ChIP-qPCR measures endogenous enhancer activity in this particular chromatin context. Together, these findings support our hypothesis that local allele-specific enhancer activities are influenced by global haplotype structure due to chromatin looping interactions.

Our chromatin interaction data also revealed that *BLK* promoter interacts haplotype-specifically with a distal repressor REST binding site and the *FAM167A* gene promoter, with the interactions being stronger on the SLE risk haplotype (Figures 2,3), consistent with reduced expression of *BLK* on the risk haplotype, and the hypothesis that *BLK* promoter is “enhancer-like” and may regulate expression of *FAM167A,* respectively. For recent research on transcriptional regulation by “enhancer-like” promoters, see (Xu et al., 2011; Dao et al., 2017; Dao and Spicuglia, 2018). We tested our hypothesis on the regulation of *FAM167A* expression by the “enhancer-like” *BLK* promoter using dCas9-KRAB chromatin repressor experiments (Figure 4). These experiments have provided strong evidence in support of our hypothesis.

These results nominate *FAM167A*, in addition to *BLK*, or both as potential risk genes at the *FAM167A-BLK* locus. If the regulatory structure we present herein is responsible for the change in disease risk for SLE, and maybe, other disorders associated with the *FAM167A-BLK* locus, rather than a different regulatory structure in another cell type, then perhaps *FAM176A* is the true gene mediating disease risk for two reasons. First, down-regulating signal transduction from the B cell receptor by reducing *BLK* expression would seem to be counter-intuitive for a disease like SLE where autoantibody generation is central to pathogenesis. Second, DIORA-1 (the gene product of *FAM167A*) has an activity that could well have a profound influence on the inflammatory response. DIORA-1 is a disordered protein (Mentlein et al., 2018) that is secreted and binds desmoglein-1 (DSG1) to gain cell entry, which then activates NFκB via its non-canonical pathway by liberating NFκB-inhibitor kinase from DSG1. DIORA-1 appears responsible for much BCR-ABL-tyrosine kinase inhibitor resistance in chronic myelogenous leukemia (CML) (Yang et al., 2022). Certainly, DIORA-1 has functional properties that make the level of activity of this gene product attractive for mediating SLE risk. An understanding of the evolutionary advantage for *BLK* and *FAM167A* to be reciprocally regulated in the way we describe awaits a deeper understanding of the inter-relationships of the pathways impacted by their two gene products. However, the multiple diseases with risk variants at this locus would be consistent with a continuing evolutionary impact.

Haplotype-specific enhancer-enhancer and enhancer-promoter chromatin interactions (Figure 2), and evidence for the “local enhancer activity amplification due to long-range chromatin interactions” hypothesis (Figure 5) have led us to seek functional evidence of synergistic interactions of *BLK* transcriptional regulatory elements. Using dCas9-p300 CRISPR epigenome editing experiments, we have activated the silent *BLK* locus in K562 cells and demonstrated enhancer-enhancer and enhancer-promoter synergies in *BLK* activation (Figure 6).

Our genetic analyses have revealed that enhancer haplotypes can modulate *BLK* expression (Figure 7) and suggested a “risk dosage” model whereby disease risk alleles at multiple regulatory elements at *BLK* locus synergistically decrease gene expression, and consequently, increase disease risk.

These studies have been done with LCLs, which are generally, B cell lines that have been infected by Epstein-Barr virus (EBV), which is strong etiologic candidate for causing SLE and Multiple Sclerosis (MS) (Harley and James, 2006; Bjornevik et al., 2022; Laurynenka et al., 2022). The LCL is a stable transformed cell line expressing the Latency III program of EBV. The EBV gene product and transcription co-factor, EBNA2, is concentrated at SLE and MS risk loci, including *BLK* (Harley et al., 2018, p. 2; Yin et al., 2021). Recently, Afrasiabi et al. (2022), have extended these observations by showing that *BLK* and *FAM167A* are bound by EBNA2 and the products of both genes are differentially expressed as eQTLs in greater magnitude in LCLs than in B cells that are not EBV infected, with *BLK* and *FAM167A* being affected in opposing directions. Both *BLK* and *FAM167A* are correlated with EBV DNA copy number per cell, with the association with *FAM167A* being much more convincing. Finally, the level of EBNA2 in their data is inversely proportional to the level of *BLK* expression. These observations add another level of complexity relating the environment to disease risk that begs for an understanding of how these differences may or may not be components of mechanisms that influence disease risk.

Common SNPs with effect sizes well below genome-wide statistical significance account for a large proportion of “missing heritability” of many traits (Yang et al., 2010). However, mechanistic details of how weak-effect genetic variants contribute to heritability and disease risk remain largely unknown because we still have very limited knowledge of how these variants percolate through the entire cellular and gene regulatory networks (Boyle et al., 2017). Investigations such as those in (Corradin et al., 2016; Boyle et al., 2017), and the present study represent important steps toward deciphering the mechanistic details of the genotype-phenotype map in disease etiology.

## Data Availability

The high-throughput sequencing data from this study have been submitted to the NCBI Gene Expression Omnibus (GEO; https://www.ncbi.nlm.nih.gov/geo) under accession number GSE211246. The original contributions presented in the study are included in the article/Supplementary Material. Further inquiries can be directed to the corresponding authors.
