# Supplementary material for: Haplotype-specific chromatin looping reveals genetic interactions of regulatory regions modulating gene expression in 8p23.1
Source: Front Genet. 2022 Sep 7;13:1008582. doi: 10.3389/fgene.2022.1008582 (PMC9490475; doi:10.3389/fgene.2022.1008582)
Supplement: Supplementary file 4 [file DataSheet1.PDF]

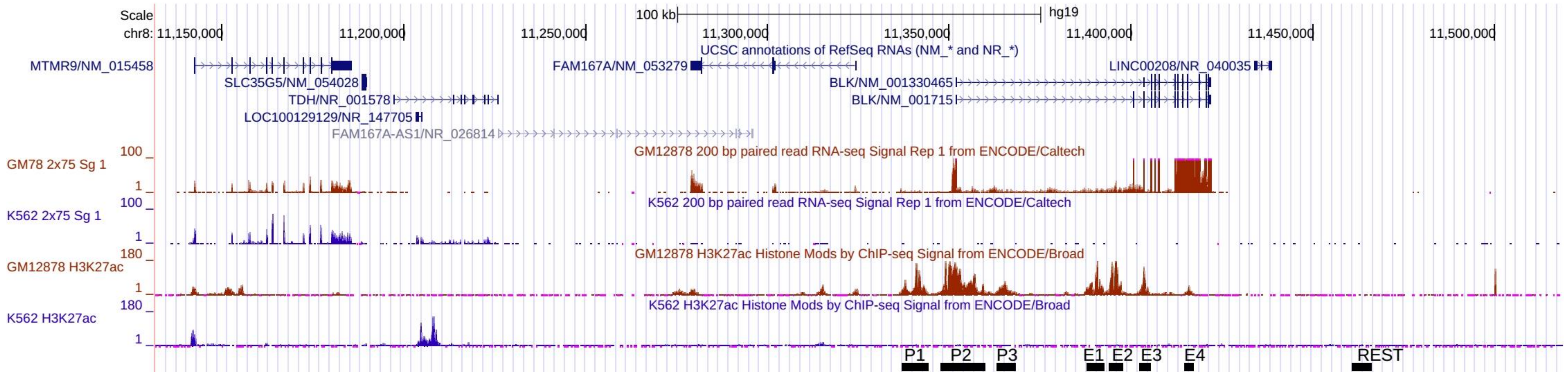

Supplementary Figure 1. RNA-seq and H3K27ac ChIP-seq signals in the vicinity of the *FAM167A-BLK* locus in GM12878 LCL and K562 cells. UCSC genome browser (http://genome.ucsc.edu/; hg19 human genome) tracks of publicly available RNA-seq and ChIP-seq data from the ENCODE project. The *BLK* and *FAM167A* genes are expressed in GM12878 LCLs, but not in K562 cells. H3K27ac, a marker of active enhancers, is highly enriched at the *BLK* promoter and putative downstream enhancers (E1, E2, E3, and E4) in LCLs and is completely depleted at these regions in K562 cells. By contrast, the *MTMR9* gene located upstream of the *FAM167A-BLK* locus is equally expressed in LCLs and K562 cells, and the H3K27ac is enriched at the promoter of *MTMR9* gene in these cells. The bottom track depicts the locations of regulatory regions used in analyses in this article (see **Supplementary Table S7**).
